# The effects of social integration and hometown identity on the life satisfaction of Chinese rural migrants: the mediating and moderating effects of a sense of belonging in the host city

**DOI:** 10.1186/s12955-020-01415-y

**Published:** 2020-06-06

**Authors:** Hongsheng Chen, Zhenjun Zhu, Jiang Chang, Yinbao Gao

**Affiliations:** 1 School of Architecture, Southeast University, No.2, Sipailou Road, Xuanwu District, Nanjing, 210096 China; 2 College of Automobile and Traffic Engineering, Nanjing Forestry University, No.159 Longpan Road, Xuanwu District, Nanjing, 210037 China; 3 Department of Geography, Environment, and Spatial Sciences, Global Urban Studies Program, Michigan State University, East Lansing, MI USA; 4 School of Architecture and Urban Planning, Chongqing University, Chongqing, 400030 China

**Keywords:** Social integration, Hometown identity, Life satisfaction, Sense of belonging, Internal migration, Rural migrant, Urbanisation

## Abstract

**Background:**

Many developing countries are rapidly urbanising because of large migration flows from rural to urban areas. However, migrants’ socio-cultural transitions might be challenging, and their extent of social integration and sense of hometown identity might impede resettlement and quality of life. Although a sense of belonging in the host city and a sense of attachment to the hometown might be important to migrants’ ability to assimilate, adapt and succeed in a new place, these factors have not adequately been studied in China.

**Methods:**

The data were derived from the 2014 China Migrants Dynamic Survey, a large-scale questionnaire survey of Chinese migrants. This study’s sample comprised 15,990 individuals in eight Chinese cities. Life satisfaction was measured with the Satisfaction with Life Scale, and the key independent variables (social integration, sense of hometown identity and sense of belonging in the host city) were measured using multi-item constructs. Cronbach’s alpha coefficients on the four main variables were 0.76 to 0.90. A multivariable linear regression analysis was applied to a path model that assessed the mediating and moderating effects of sense of belonging in the host city on direct relationships with life satisfaction (social integration ⇢ life satisfaction and hometown identity ⇢ life satisfaction).

**Results:**

Social integration positively related to life satisfaction (B = 0.267) and sense of belonging in the host city (B = 0.912). A weak hometown identity related to higher life satisfaction and stronger sense of belonging in the host city (B = 0.087 and B = 0.176, respectively). Sense of belonging in the host city significantly mediated the relationships between social integration and life satisfaction (B = 0.267 decreased to B = 0.085) and between hometown identity and life satisfaction (B = 0.087 decreased to B = 0.048). Last, sense of belonging in the host city significantly moderated the relationship between social integration and life satisfaction.

**Conclusion:**

A sense of belonging to a place is important to migrants’ life satisfaction regarding the hometown and the host city. Promoting migrants’ sense that they are part of the new living environment is important to China’s sustainable urban development.

## Background

Life satisfaction is an important indicator for measuring migrants’ quality of life. Many scholars have studied internal migrations in developing countries, such as China, and the life satisfaction of those individuals and families [1–3]. Previous studies on migrants’ life satisfaction mostly focused on the influences of their socioeconomic status [4–6], usually finding positive associations between measures of socioeconomic status and life satisfaction [7, 8]. For example, Liu and colleagues [5] found that, in Guangzhou, China, migrants’ sense of relative deprivation negatively influenced their life satisfaction, and their subjective wellbeing was lower than that of the native residents. Huang et al. [4] found that objective socioeconomic status positively and significantly related to Chinese rural-to-urban migrants’ subjective wellbeing. The social relationships that migrants develop with their host neighbourhoods and cities also might be important to their life satisfaction because factors at the neighbourhood, city or country level, such as social ties, influenced migrants’ life satisfaction [9, 10]. Net of the influences of socioeconomic factors, the extent of assimilation into the host culture was positively related to migrants’ life satisfaction [11]. Chen et al. [12] found that migrants were more satisfied than native residents in cities with populations of 200,000 to 500,000 residents. Kogan et al.’s [13] results suggest that migrants are more likely to be satisfied in countries with welcoming social contexts. Overall, the more assimilated, adapted or integrated migrants are to their host country’s social environment, the higher their life satisfaction.

However, few previous studies have focused on the link between migrants’ sense of hometown identity and their life satisfaction after they resettle in the city. Hometown identity is a kind of social identity, and its formation is mainly affected by a resident’s living habits, behavioural norms, values, and emotional belonging formed when they lived in their hometown. However, the impact of hometown identity on the physical and mental health of migrants living in new places is controversial. For example, Elahi et al. [14] found that hometown identities do not provide psychological protection from developing mental health symptoms associated with financial stress. Nonetheless, some studies also found that a hometown identity can lead to migrants receiving psychological and economic support from their fellow migrants, which is favourable for them to adapt to the new environment [15, 16]. Furthermore, important lifestyle and standard of living differences between migrants’ hometowns and their host community, such as customs, diet, ways of socially interacting, and social norms may exist, and the hometown culture might not conform to the characteristics of the new environment. Difficulties related to changing the hometown identity might create obstacles to assimilation, adaptation and integration into the host city, which might, in turn, negatively influence life satisfaction [11]. In China, cities are strongly attracting rural residents because of the large gap in development between urban and rural places, and large numbers of rural individuals and families have been migrating to cities in the past decades [17–19]. Although many rural migrants move to cities in search of a better life, not all are able to achieve their aspirations [20, 21]. Many rural migrants’ living and working conditions are worse than those of the native residents of their host cities [22, 23], which may negatively influence their sense of belonging and life satisfaction in the city. In this context, a sense of belonging in the host city might significantly influence migrants’ life satisfaction. A sense of belonging might moderate or mediate the influences of environmental factors on life satisfaction. The previous studies do not clearly address these possibilities, and, to fill that gap, this study analysed the influences of a sense of belonging in the host city on the relationship between migrants’ social integration and their life satisfaction and between their sense of hometown identity and life satisfaction.

**Fig. 1:**
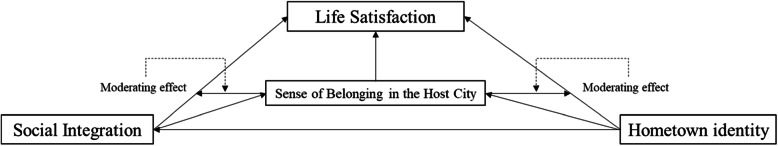
The conceptual framework illustrating the hypothesised relationships tested in the study

Based on the previous literature, we developed a conceptual framework and path model (Fig. 1) with two possible paths by which migrants’ social integration might influence their life satisfaction. First, we expected social integration directly (and positively) to influence life satisfaction, and we expected that relationship to be moderated (by increasing its strength) by the sense of belonging in the host city. Second, we expected the sense of hometown identity directly (and negatively) to relate to life satisfaction, and we expected the sense of belonging in the host city to mediate and moderate (weakening its effect) that relationship. As Fig. 1 illustrates, all three key independent variables were hypothesised to directly influence life satisfaction, sense of hometown identity was expected to (negatively) influence social integration (but not vice versa), and the sense of belonging in the host city was the key intervening variable.

## Methods

### Data

This study used data derived from the China Migrants Dynamic Survey 2014 (CMDS 2014) to test the path model illustrated in Fig. 1 above. The CMDS 2014 was a large-scale survey of migrants in China conducted by the National Health Commission of the People’s Republic of China. We analysed data collected in eight Chinese cities. The eight cities include: Beijing, China’s capital; Qingdao, Xiamen, Shenzhen, the most economically developed cities in Shandong, Fujian and Guangdong provinces, respectively; Zhengzhou and Chengdu, the capitals of Henan and Sichuan provinces, respectively; Jiaxing and Zhongshan, two cities with large floating populations. After dropping all the cases with missing data, the sample comprised valid data on 15,990 internal migrants. A multivariable linear regression model was used to estimate the influences of the independent variables on life satisfaction.

### Variables

#### Dependent variable

To measure life satisfaction, the CMDS used the Satisfaction With Life Scale, which asks for the extent of agreement with five items: (1) ‘In most ways my life is close to my ideal’, (2) ‘The conditions of my life are excellent’, (3) ‘I am satisfied with my life’, (4) ‘So far I have gotten the important things I want in life’, and (5) ‘If I could live my life over, I would change almost nothing’. The response options were on a seven-point scale where 1 = *strongly disagree* through 7 = *strongly agree*. The responses on the five items were summed for a total score ranging from five to 35, and Cronbach’s reliability coefficient was alpha = 0.86. In this study, the dependent variable is in the logarithmic form was used in the regression models.

#### Main independent variables

The main independent variables were social integration, sense of hometown identity, and sense of belonging in the host city. Social integration is a broad concept that is mainly used to describe the degree of residents’ integration into a new social environment. In this study, we selected items on social interaction and neighbourhood identity from the database provided by CMDS to construct social integration variables. We defined hometown identity as migrants being influenced by the living and behavioural habits of their hometowns. Therefore, in CMDS, items related to migrants’ hometown habits were selected to measure their degree of hometown identity. Sense of belonging in the city is used to evaluate the psychological connection between migrants and the city. The independent variables of social integration, sense of hometown identity, and sense of belonging in the host city are in the logarithmic form used in regression models.

##### Social integration

Social integration was measured using responses to 10 items with response options of 1 = *strongly disagree* through 4 = *strongly agree*: ‘How much do you agree that: (1) you are willing to live in the same neighbourhood as native residents, (2) you are willing to work with native residents, (3) you are willing to be a neighbour of native residents, (4) you are willing to be friends with native residents, (5) you would marry or accept a family member marrying a native resident, (6) you are willing to integrate into your new neighbourhood, (7) the native residents are willing to accept you as a member of the community, (8) the native residents are willing to be your neighbours, (9) the native residents like you, and (10) the native residents respect you’. The Cronbach’s reliability coefficient of the 10 items was alpha = 0.87, and the sum of the scores on the 10 items was used as a total score to measure the extent of social integration. Higher scores indicated more integration and the variable ranged from 10 through 40.

##### Sense of hometown identity

Eight items were used to assess the extent to which the respondents thought it was important to maintain aspects of their hometown identity: ‘It is important that: (1) I keep the customs of my hometown, (2) I keep the social norms of my hometown, (3) I teach my children to speak my hometown’s language, (4) I keep my hometown’s lifestyle, such as eating habits, (5) my hygiene habits are quite different from those of the native residents, (6) my clothing is quite different from that of the native residents, (7) my ideas about education and pensions are quite different from that of the native residents, and (8) my views on some social issues are quite different from those of the native residents. Agreement was rated on a scale of 1 = *strongly agree* to 5 = *strongly disagree*. The scores on the eight items were summed for a composite score with a range of eight through 40, higher scores indicated a weaker sense of hometown identity and Cronbach’s reliability coefficient was 0.76 on the eight items.

##### Sense of belonging in the host city

Three items were used to assess the respondents’ sense of belonging in the host city. They were asked to indicate their agreement on a scale where 1 = *strongly disagree* through 4 = *strongly agree* regarding: (1) ‘I belong in this city’, (2) ‘I am a member of this city’, and (3) ‘I am a part of this city’. The scores on the three items were summed for a total score ranging from three through 12, higher scores indicated a stronger sense of belonging in the host city and Cronbach’s reliability coefficient was 0.90.

#### Control variables

Because previous studies found significant associations between socioeconomic status and life satisfaction, we controlled for the effects of socioeconomic factors in the analysis. Age (in years), gender, self-rated physical health (scores 1–5, 1 for ‘very poor health’ to 5 for ‘very good health’), (log) total household monthly income (amounts), homeownership in the host city (yes or no), occupation (type), educational attainment (degree category), length of residence in the host city (in years), and hours per day working during the past month (in hours) were all used as covariates.

## Results

Table 1 presents the descriptive statistics of all the variables used in the analysis. Table 2 shows the main regression results. The dependent variable in Models 1, 3, 4, and 6 was life satisfaction, and the dependent variable in Models 2 and 5 was sense of belonging in the host city. The Model 1 results found that, net of the effects of the nine control variables, social integration positively and significantly related to life satisfaction (coefficient = 0.267). Age, health and monthly household income positively and significantly related to life satisfaction (*p* < .01). Females had higher life satisfaction than males (*p* < .01), and homeowners had higher life satisfaction than non-homeowners (*p* < .01). Civil servants, clerks and self-employed respondents had higher life satisfaction (*p* < .01) and unemployed respondents had lower life satisfaction (*p* < .01) than service or manufacturing workers. Respondents with senior high school (*p* < .05) or college or more (*p* < .01) education had lower life satisfaction than those with junior high school or less education. Working hours inversely related to life satisfaction (*p* < .01).

**Table 1:** Descriptive statistics of the variables used in the analysis (*n* = 15,990)

Variables	Mean (SD) / Percentage
Life satisfaction (rang: 5–35)	21.85 (6.25)
Social integration (range: 10–40)	33.47 (4.31)
Sense of hometown identity (range: 8–40)	23.79 (4.09)
Sense of belonging in the host city (range: 3–12)	9.63 (1.86)
Age (in years) (range: 15–60)	32.69 (8.72)
Gender (%)	
Female	45
Male	55
Self-rated physical health (range: 1–5)	3.76 (0.97)
Total household monthly income (CNY)	6433.49 (7052.23)
Homeownership in the host city (%)	
Yes	9.13
No	90.87
Occupation (%)	
Service or manufacturing	66.91
Administration, management, professional, or technician	7.75
Civil service, clerical, or self-employed	15.76
Unemployed	9.58
Education (%)	
Junior high school or less	59.95
Senior high school	25.32
College or more	14.73
Length of residence in the host city (in years) (1–42)	5.26 (4.43)
Hours per day spent on work in last month (in hours) (0–16)	8.85 (2.78)

**Table 2:** The relationships of social integration and sense of hometown identity to life satisfaction and the mediation effects of sense of belonging in the host city on those relationships controlling for the effects of nine control variables (*n* = 15,990)^a^

Independent variables	Model 1: Life satisfaction		Model 2: CB		Model 3: Life satisfaction		Model 4: Life satisfaction		Model 5: CB		Model 6: Life satisfaction	
	B	SE	B	SE	B	SE	B	SE	B	SE	B	SE
(Log)SI	0.2674***	0.0192	0.9137***	0.0104	0.0852***	0.0232						
(Log)HI							0.0871***	0.0138	0.1762***	0.0090	0.0477***	0.0138
(Log)CB					0.1994***	0.0145					0.2233***	0.0121
**Control variables**												
Age	0.0030***	0.0003	0.0004**	0.0002	0.0030***	0.0003	0.0033***	0.0003	0.0012***	0.0002	0.0031***	0.0003
Gender (ref: male)	0.0246***	0.0053	0.0025	0.0029	0.0241***	0.0053	0.0259***	0.0053	0.0072**	0.0035	0.0242***	0.0053
Self-rated health	0.0573***	0.0027	0.0054***	0.0015	0.0563***	0.0027	0.0639***	0.0027	0.0272***	0.0017	0.0578***	0.0027
(Log) household monthly income	0.0725***	0.0048	−0.0026	0.0026	0.0730***	0.0048	0.0711***	0.0048	−0.0077**	0.0031	0.0728***	0.0048
Homeowner (ref: no)	0.0669***	0.0094	0.0435***	0.0051	0.0582***	0.0094	0.0734***	0.0095	0.0726***	0.0061	0.0572***	0.0094
Occupation (ref: service or manufacturing)												
Administration	−0.0017	0.0102	0.0114**	0.0055	−0.0040	0.0102	0.0019	0.0103	0.0254***	0.0067	−0.0038	0.0102
Civil service	0.0258***	0.0074	0.0122***	0.0040	0.0234***	0.0074	0.0288***	0.0075	0.0237***	0.0048	0.0235***	0.0074
Unemployed	−0.0276**	0.0112	− 0.0127**	0.0061	− 0.0251**	0.0111	− 0.0291***	0.0113	− 0.0205***	0.0073	− 0.0245**	0.0112
Education (ref: junior high school or less)												
Senior HS	−0.0149**	0.0062	0.0109***	0.0034	−0.0171***	0.0062	−0.0167***	0.0063	0.0077*	0.0041	−0.0184***	0.0062
College or more	−0.0327***	0.0081	0.0029	0.0044	−0.0333***	0.0080	−0.0333***	0.0081	0.0072	0.0053	−0.0349***	0.0080
Length of residence	0.0003	0.0006	0.0008**	0.0003	0.0001	0.0006	0.0006	0.0006	0.0019***	0.0004	0.0002	0.0006
Hours per day worked during the past month	−0.0058***	0.0012	−0.0026***	0.0006	−0.0053***	0.0012	−0.0058***	0.0012	−0.0026***	0.0008	−0.0052***	0.0012
Constant	1.2018***	0.0788	−0.9590***	0.0427	1.3931***	0.0795	1.8384***	0.0622	1.6074***	0.0404	1.4794***	0.0645
R^2^	0.084		0.350		0.094		0.075		0.060		0.094	
Adj. R^2^	0.083		0.350		0.094		0.074		0.060		0.093	
Log likelihood	− 4615.0624		5160.5506		− 4520.8870		− 4691.6582		2209.8000		− 4521.6515	

* = *p* < .10, ** = *p* < .05, *** = *p* < .01

^a^*SI* Social integration, *HI* Sense of hometown identity, *CB* Sense of belonging in the host community, *SE* Standard Error, *HS* high school

In Model 2, the dependent variable was sense of belonging in the host city. Social integration had a positive influence net of the effects of the control variables (coefficient = 0.912). Positive effects were found for age (*p* < .05), self-rated health (*p* < .01), length of residence (*p* < .05), and working hours (*p* < .01) was negatively related to sense of belonging in the host city. Homeowners had a stronger sense of belonging in the host city than non-homeowners (*p* < .01), and respondents with a senior high school education had a stronger of belonging in the community than those who had less education (*p* < .01). The service or manufacturing workers had a stronger sense of belonging in the host city than the unemployed respondents (*p* < .05), but a weaker sense than the administration group (*p* < .05) or the civil service group (*p* < .01).

Model 3 estimated the influences of social integration and sense of belonging in the host city on life satisfaction and found that both were positively and significantly associated with life satisfaction (B = .085 and B = .199, respectively). The influences of the nine covariates on life satisfaction were very similar to their effects found in Model 1. Model 4 (dependent variable was life satisfaction) and Model 5 (dependent variable was sense of belonging in the host city) focused on the influence of sense of hometown identity net of the influences of the control variables, which was positive and significant in both models (Model 4 coefficient = 0.087; Model 5 coefficient = 0.176). In other words, respondents with a weaker sense of hometown identity had higher life satisfaction and a stronger sense of belonging in the host city. The influences of the covariates on life satisfaction in Model 4 were very similar to their effects in Model 3. However, whereas there were no income or gender differences in Model 2, in Model 5, household income was inversely related (*p* < .05) and females had a stronger sense of belonging than males (*p* < .05). Model 6 was the final estimation of effects on life satisfaction. A sense of hometown identity and sense of belonging in the host city had positive and significant effects on life satisfaction (B = 0.048 and B = 0.223, respectively) net of the effects of the covariates, and the covariates’ influences were similar to those of Models 1, 3 and 4.

**Fig. 2:**
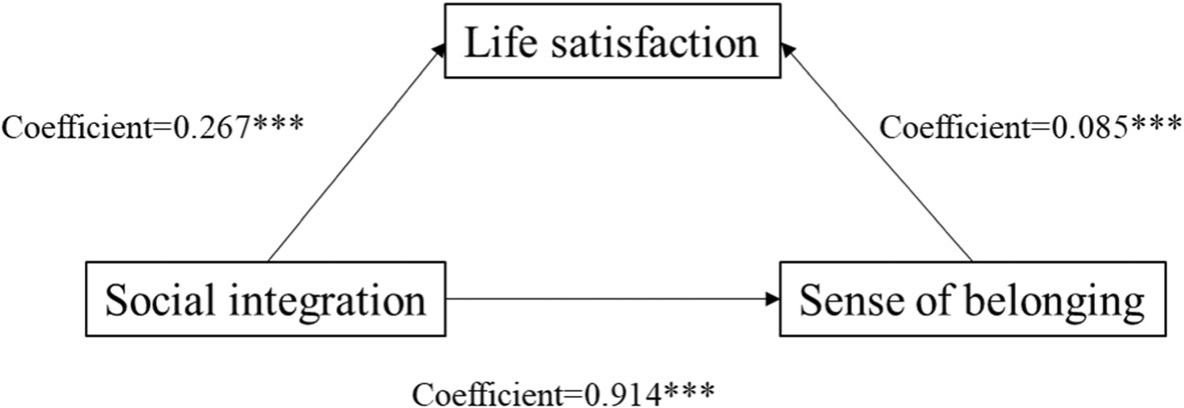
Unstandardized coefficients estimating social integration ⇢ sense of belonging in the host city ⇢ life satisfaction; * = *p* < .10, ** = *p* < .05, *** = *p* < .01

**Fig. 3:**
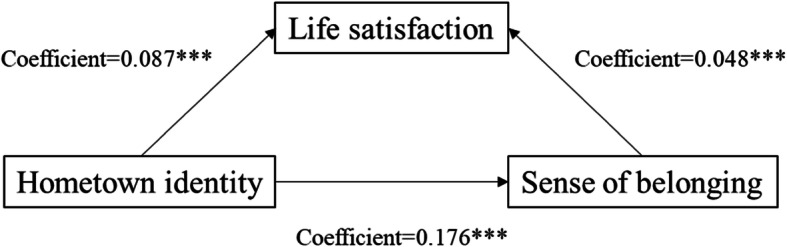
Unstandardized coefficients estimating hometown identity ⇢ sense of belonging in the host city ⇢ life satisfaction; * = *p* < .10, ** = *p* < .05, *** = *p* < .01

**Fig. 4:**
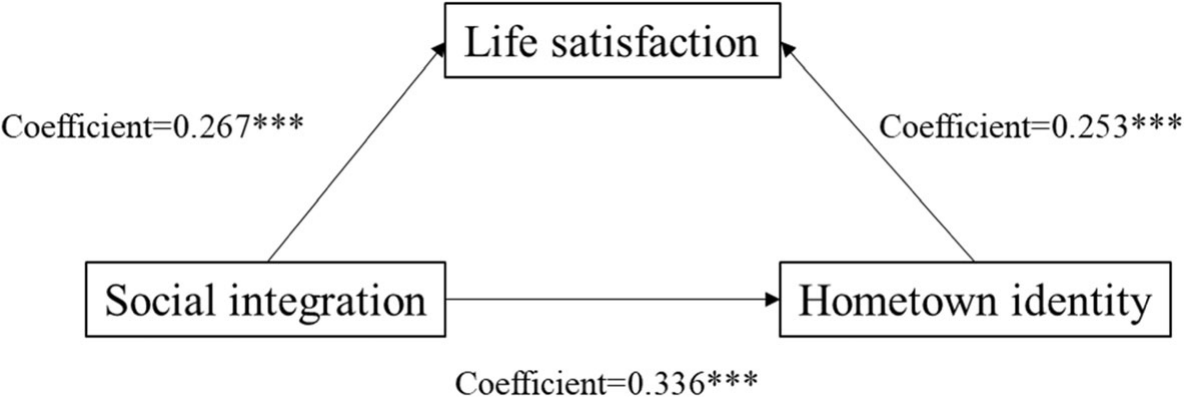
Unstandardized coefficients estimating the path of social integration ⇢ sense of hometown identity ⇢ life satisfaction; * = *p* < .10, ** = *p* < .05, *** = *p* < .01

We followed Baron and Kenny [24] to test the presence of mediation effects of the sense of belonging in the host city. Figures 2 and 3 show the results of Model 3 indicating that adding the measure of sense of belonging in the host city to Model 1 weakened the effect of social integration on life satisfaction from B = 0.267 to B = 0.085. The results of Model 6 (in Table 2) indicated that, when the measure of the sense of belonging in the host city was added to Model 4, the influence of sense of hometown identity on life satisfaction decreased from B = 0.087 to B = 0.048. Therefore, we concluded that sense of belonging in the host city significantly mediated the relationship between social integration and life satisfaction. Table 3 and Fig. 4 show that sense of hometown identity also significantly influenced the relationship between social integration and life satisfaction.

**Table 3:** The effect of social integration on hometown identity (*n* = 15,990)

Variable	Hometown identity		Life satisfaction	
	B	Standard error	B	Standard error
(Log) social integration	0.3363***	0.0107	0.2528***	0.0198
(Log) hometown identity			0.0436***	0.0141
**Control variables**				
Age	−0.0018***	0.0002	0.0031***	0.0003
Gender (ref: male)	0.0026	0.0030	0.0245***	0.0053
Self-rated physical health	−0.0131***	0.0015	0.0579***	0.0027
(Log) total household monthly income	−0.0007	0.0027	0.0725***	0.0048
Homeowner (ref: no)	0.0429***	0.0053	0.0650***	0.0094
**Occupation (ref: service or manufacturing)**				
Administration, management, professional, or technician	0.0082	0.0057	−0.0021	0.0102
Civil service, clerical, or self-employed	0.0060	0.0042	0.0256***	0.0074
Unemployed	−0.0194***	0.0063	−0.0268**	0.0112
**Education (ref: junior high school or less)**				
Senior high school	0.0251***	0.0035	−0.0160**	0.0063
College or more	0.0468***	0.0045	−0.0348***	0.0081
Length of residence	0.0003	0.0004	0.0003	0.0006
Hours per day working during the past month	−0.0005	0.0007	−0.0057***	0.0012
Constant	2.0747***	0.0440	1.1114***	0.0840
R^2^	0.087		0.084	
Adj. R^2^	0.086		0.083	
Log likelihood	4677.6046		− 4610.3117	

* = *p* < .10, ** = *p* < .05, *** = *p* < .01

The moderating effect of sense of belonging in the host city was tested and the results are shown in Table 4. The sense of belonging had a significant moderating effect on the relationship between social integration and life satisfaction. That is, in the same degree of social integration, migrants with a strong sense of belonging have higher life satisfaction than those with a weak sense of belonging, which shows that the negative effect of low social integration on life satisfaction was mitigated among those with a strong sense of belonging in the host city.

**Table 4:** The moderating effect of a sense of belonging in the host city on the relationship between social integration and life satisfaction (*n* = 15,990)

Variable		Life satisfaction	
		B	Standard error
(Log) Social integration		−0.1910	0.3428
(Log) Hometown identity		0.0227	0.3035
(Log) Sense of belonging		−0.8552***	0.2495
(Log) Social integration × (Log) Hometown identity		−0.0616	0.1070
(Log) Hometown identity × (Log) Sense of belonging		0.1038	0.0669
(Log) Social integration × (Log) Sense of belonging		0.2119***	0.0523
**Control variables**			
Age		0.0030***	0.0003
Gender (ref: female)		0.0238***	0.0053
Self-rated physical health		0.0557***	0.0027
(Log) Total household monthly income		0.0732***	0.0048
Homeowner (ref: no)		0.0535***	0.0094
Occupation (ref: service or manufacturing)			
Administration, management, professional or technician		−0.0056	0.0102
Civil service, clerical or self-employed		0.0225***	0.0074
Unemployed		−0.0242**	0.0111
Education (ref: junior high school or less)			
Senior high school		−0.0184***	0.0062
College or more		−0.0357***	0.0080
Length of residence		0.00004	0.0006
Hours per day spent working during the past month		−0.0052***	0.0012
Constant		2.9320***	0.9514
R^2^ = 0.096	Adj. R^2^ = 0.095	Log likelihood = − 4504.5677	

* = *p* < .10, ** = *p* < .05, *** = *p* < .01

## Discussion

The results of this study support previous studies [13, 25, 26] that found migrants with more social integration were more satisfied with their lives. However, we found that the stronger the hometown identity, the lower the life satisfaction. Many previous studies have suggested that migrants change their sense of place-related identity and lifestyle after relocating and integrating into the host city [27], which should increase their life satisfaction. Previous studies have suggested that migrants with strong hometown identities might have problems with social integration into a new community [28, 29]. We found that social integration had a direct influence on hometown identity, which, in turn, influenced life satisfaction. These results imply that migrants’ sense of hometown identity might progressively weaken as they integrate into their new communities, which was not emphasised by previous studies.

We proposed that a sense of belonging in the host city is an important influence on migrants’ life satisfaction. In many developing countries, rural development lags behind urban growth and advancements and a popular saying in China is ‘*cheng shi rang sheng huo geng mei hao*’, which means ‘the city makes life better’, which reflects the desire that rural people have for city life. We found that a sense of belonging in the host city had a positive, significant and direct influence on life satisfaction, and it had significant mediating influences on the relationship between social integration and life satisfaction and on the relationship between hometown identity and life satisfaction.

The extent of migrants’ social integration and the strength of their sense of hometown identity influenced the sense of belonging in the host city, and, in turn, life satisfaction. In China, the differences between urban and rural areas means that migrants from rural and/or underdeveloped areas might find it difficult to adapt to urban lifestyles. If they never adapt to city life, their sense of belonging there might be very weak. We found that a sense of belonging in the host city weakened the negative influence of low social integration on life satisfaction. This finding also enhances our understanding of the reasons that migrants stay in the city when they do not quickly integrate into their communities. We have tended to assume that migrants move to cities for financial reasons, such as higher incomes [30], but this study found that the desire for city life was a reason for migrating. Having strong positive expectations about the future in a city is an important reason why they can endure lack of integration while they live there.

China is rapidly urbanising and rural emigrants are continually flowing into urban areas in eastern China. It is a difficult social transition for the migrants. Regarding public health policy, we propose that it is necessary to consider the influences of the extents of social integration and strength of hometown identities on migrants’ life satisfaction. Second, many migrants are considered to be outsiders in their host cities, and they face various types of discrimination that might negatively influence their sense of belonging there. Thus, urban planners and designers should give more attention to migrants’ needs. Third, the Chinese government should establish public services targeted to meet migrants’ physical and mental health needs and to help them experience smooth integration into their new communities and to improve their life satisfaction.

## Conclusion

This study employed data derived from a large-scale questionnaire survey to examine migrants’ life satisfaction. It provides evidence that Chinese migrants with relatively high social integration had higher life satisfaction than those with lower social integration. A sense of belonging in the host city was a positive and significant influence on life satisfaction. However, a strong sense of hometown identity was associated with lower life satisfaction and a weaker sense of belonging in the host city. The sense of belonging in the host city had a significant mediating role in the relationship between social integration and life satisfaction and in the relationship between hometown identity and life satisfaction. Last, the influence of social integration on life satisfaction was mitigated by the sense of belonging in the host city.

## Data Availability

Data used in this study were derived from 2014 China Migrants Dynamic Survey (CMDS 2014), which is a large-scale sample survey of migrants in China conducted by the National Health Commission of the People’s Republic of China. The opinions in this paper are those of the authors.
